# Electron Capture Dissociation and Collision-Induced Dissociation of Metal Ion (Ag^+^, Cu^2+^, Zn^2+^, Fe^2+^, and Fe^3+^) Complexes of Polyamidoamine (PAMAM) Dendrimers

**DOI:** 10.1016/j.jasms.2008.12.013

**Published:** 2009-04

**Authors:** Malgorzata A. Kaczorowska, Helen J. Cooper

**Affiliations:** School of Biosciences, College of Life and Environmental Sciences, University of Birmingham, Edgbaston, Birmingham, United Kingdom

## Abstract

The electron capture dissociation (ECD) and collision-induced dissociation (CID) of complexes of polyamidoamine (PAMAM) dendrimers with metal ions Ag^+^, Cu^2+^, Zn^2+^, Fe^2+^, and Fe^3+^ were determined by Fourier transform ion cyclotron resonance mass spectrometry. Complexes were of the form [PD + M + mH]^5+^ where PD = generation two PAMAM dendrimer with amidoethanol surface groups, M = metal ion, m = 2–4. Complementary information regarding the site and coordination chemistry of the metal ions can be obtained from the two techniques. The results suggest that complexes of Fe^3+^ and Cu^2+^ are coordinated via both core tertiary amines, whereas coordination of Ag^+^ involves a single core tertiary amine. The Zn^2+^ and Fe^2+^ complexes do not appear to involve coordination by the dendrimer core.

## Graphical Abstract

Complementary information regarding the structure and coordination chemistry of metal ion complexes of PAMAM dendrimers may be obtained from ECD and CID mass spectrometry.

**Figure:**
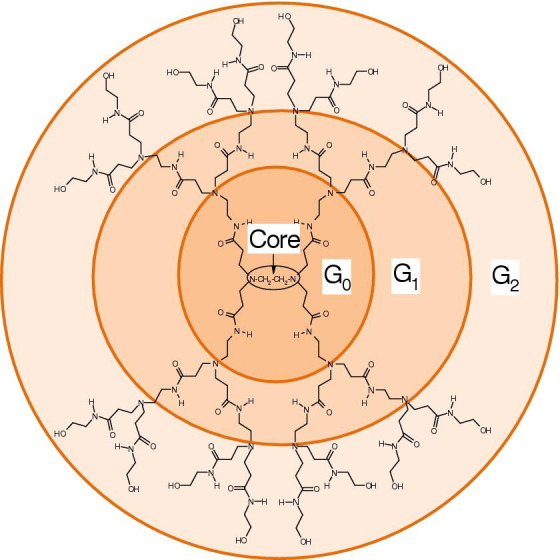

Polyamidoamine (PAMAM) dendrimers [1] are a family of polymers with tree-like structures characterized by a high degree of molecular uniformity. Their structures comprise a central core, amidoamine repeating branches, and terminal surface groups. PAMAM dendrimers allow precise control of size and mass; their structure grows linearly as a function of additional amidoamine branches (generations), and the number of surface groups increases with the number of generations [2]. The chemical and structural properties of PAMAM dendrimers make them well-suited for numerous applications including drug delivery, gene transfection, imaging [2, 3, 4, 5], and catalysis [6, 7]. PAMAM dendrimers are also used as templates for the fabrication of nanoparticles [8, 9, 10, 11, 12], and are particularly attractive as high-capacity chelating agents for metal ions [13].

Successful fabrication of metal nanoparticles inside dendrimers is a multistep process involving controlled synthesis of dendrimers, metal ion complexation [8, 14], reduction, and finally nucleation of the metal ions [12]. Complexation of metal ions is critical in determining the nucleation sites within the dendrimer. When favorable absorption sites compete, internal migration of the guest ions is observed [15]. Differences in metal ion affinities for dendrimer sites may be used for the production of bimetallic particles and the exchange of ions encapsulated by the dendrimer [16]. Encapsulation of metal ions by PAMAM dendrimers typically involves internal tertiary amine groups. In the case of low-generation PAMAM dendrimers, reactive sites are also localized at the periphery [17].

As a result of the growing interest in intradendrimer metal nanoparticles, there is a need for techniques for the characterization of both the dendrimer host structure and the corresponding metal–dendrimer complex. As described by Schalley and coworkers [18], mass spectrometry is a valuable tool for the characterization of dendrimers under environment-free conditions. The collision-induced dissociation (CID) of PAMAM dendrimers and their singly and doubly charged complexes with Ag^+^ has been investigated by Brodbelt and coworkers [19]. Electron capture dissociation (ECD) of a third generation PAMAM dendrimer was described by Oh and coworkers [20]. Previous work in our laboratory compared the ECD and CID of protonated PAMAM dendrimers of different generations and surface groups [21]. The ECD was dominated by cleavage at the tertiary amines in the innermost generations and was independent of the nature of the surface group. In contrast, CID tended to occur in the outermost generations and showed a strong dependence on surface group.

Here, we present the ECD and CID of metal ion (Cu^2+^, Fe^3+^, Ag^+^, Zn^2+^, Fe^2+^) PAMAM dendrimer complexes. The results are discussed in relation to the established ECD and CID fragmentation of protonated PAMAM dendrimers [21]. The results demonstrate that complementary information regarding location and coordination chemistry of the metal ions may be gleaned from the ECD and CID patterns.

## Experimental

### Sample Preparation

The second generation polyamidoamine (PAMAM) dendrimer with amidoethanol surface groups and ethylenediamine core was purchased from Sigma-Aldrich (Poole, Dorset, UK) and used without further purification. The metal salts: AgNO_3_, CuCl_2_^·^2H_2_O, ZnCl_2_, FeSO_4_, and FeCl_3_ were purchased from Fisher Scientific (Loughborough, UK), Sigma-Aldrich (Poole, Dorset, UK), Sigma Chemical Co. (St. Louis, MO), Sigma Chemical C. (St. Louis, MO), and BDH Ltd. (Poole, UK), respectively, and were used as received. The PAMAM dendrimer was diluted to a concentration of 1 mM in methanol (Fisher Scientific, Loughborough, UK); metal salts were dissolved in water (J. T. Baker, Deventer, Holland) to a concentration of 1 mM. Analytical solutions of PAMAM dendrimer and metal salts were prepared (1:1) in methanol: water (50:50, vol/vol). A portion of this solution was diluted in methanol:water:acetic acid (Fisher Scientific, Loughborough, UK) (49:49:2, vol/vol) to give a final concentration of 10 μM PAMAM dendrimer and 10 μM metal salt.

### Mass Spectrometry

All mass spectrometry experiments were performed on a Thermo Finnigan LTQ FT mass spectrometer (Thermo Fisher Scientific, Bremen, Germany). Samples were introduced to the mass spectrometer by an Advion Biosciences Triversa Nanomate electrospray source (Advion Biosciences, Ithaca, NY). Xcalibur 2.0 software (Thermo Fisher Scientific) was used for data acquisition and analysis. All mass spectra were acquired at a resolution of 100,000 at *m/z* 400. Precursor ions for CID and ECD were isolated in the linear ion trap. Isolation width was 10 Th. Automated gain control (AGC) was used to accumulate sufficient precursor ions (target value 1 × 10^6^). Precursor ions for ECD were transmitted to the ICR cell. Electrons were generated on the surface of an indirectly heated barium tungsten cylindrical dispenser cathode (5.1 mm diameter, situated 154 mm from the cell, 1 mm off-axis), (Heat Wave Labs, Inc, Watsonville, CA). The current across the electrode was ∼1.1 A. Precursor ions were irradiated with electrons for 70 ms. All CID experiments were performed in the front-end linear ion trap and the fragments transferred to the ICR cell for detection. CID experiments were performed with helium gas at normalized collision energy of 35%. Each ECD and CID scan consisted of five co-added microscans. The MS/MS spectra were averaged over 30 scans. All the resulting spectra were analyzed manually.

## Results and Discussion

**Scheme 1:**
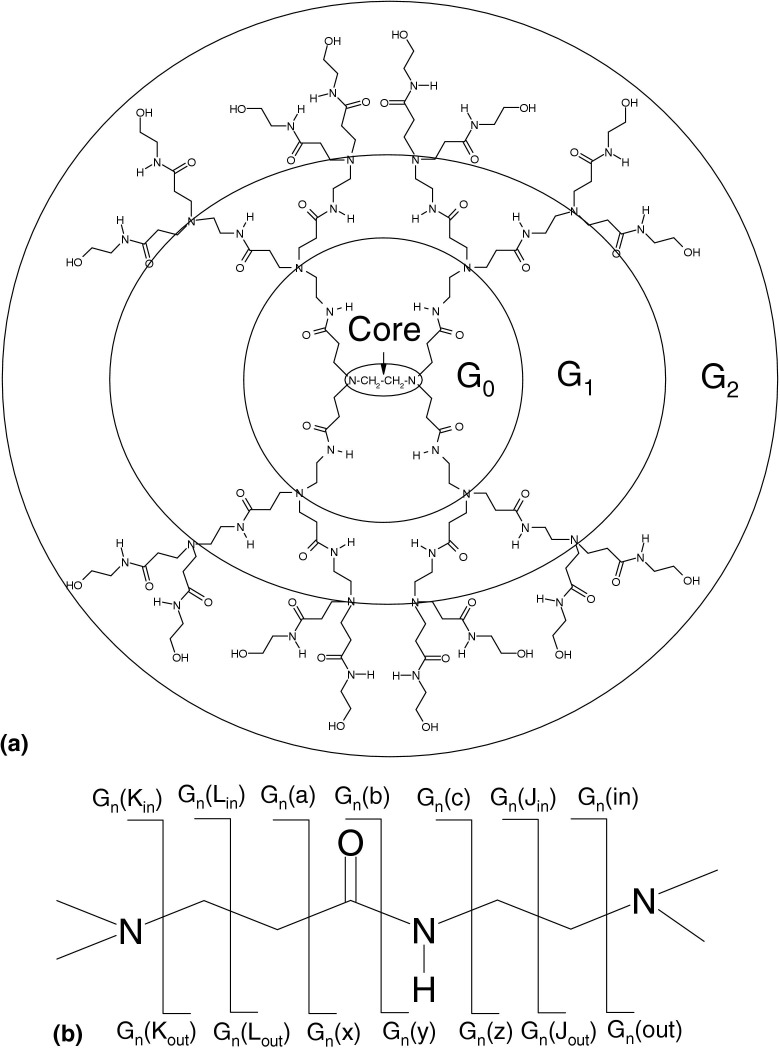
(**a**) Structure of PAMAMG2OH, second generation PAMAM dendrimer with amidoethanol surface groups and ethylenediamine core. (**b**) ECD and CID cleavage sites along the backbone of a PAMAM dendrimer.

Electrospray ionization of solutions containing equimolar concentrations of the second generation PAMAM dendrimer (ethylenediamine core, 16 amidoethanol surface groups; PAMAMG2OH) and metal ions leads to the formation of multiply-charged metal-containing ions with the general formula [PD + M^*n*+^ + mH]^(*n* + m)+^, where PD is the PAMAM dendrimer, M is the metal ion, *n* is the oxidation state of metal ion, and m is the number of protons. At higher concentrations of metal salt, complexes with the general formula [PD + 2M^*n*+^ + mH]^(2*n* + m)+^ and [PD + 3M^*n*+^ +mH]^(3*n* + m)+^ were observed, however the relative abundances of these complexes were much lower than those containing a single metal ion. The results presented here were obtained for [PD + M^*n*+^ + mH]^5+^ precursor ions, however similar fragmentation behavior (both ECD and CID) was observed for all charge states. (For comparison, the ECD and CID mass spectra of [PD + Cu^2+^ + 2H]^4+^ and [PD + Ag^+^ + 2H]^3+^ ions are shown in Supplementary Figures 1–4, which can be found in the electronic version of this article). The structure of PAMAMG2OH is shown in Scheme 1a. The notation system for representing fragmentation sites along the backbone of the PAMAM dendrimer is shown in Scheme 1b. The system is based on that proposed by Oh and coworkers [20], and follows the conventional nomenclature for peptide and protein fragment ions [22]. Assignments are given in the form G_n_(m), where subscript n refers to the generation in which fragmentation takes place and m denotes the type of fragmentation: *a*^·^*x, b/y, c*^·^/*z*^·^. G_n_(in) and G_n_(out) refer to fragmentation that takes place core-side of the tertiary amines. L and J concern cleavages between carbon atoms in the amidoamine branches, and K refers to cleavage surface-side of the tertiary amines. Fragment assignments were made on the basis of accurate mass measurement and verified by comparison of observed and expected isotope patterns.

### ECD of Complexes of PAMAMG2OH with Metal Ions: Ag^+^, Cu^2+^, Zn^2+^, Fe^2+^, and Fe^3+^

**Figure 1:**
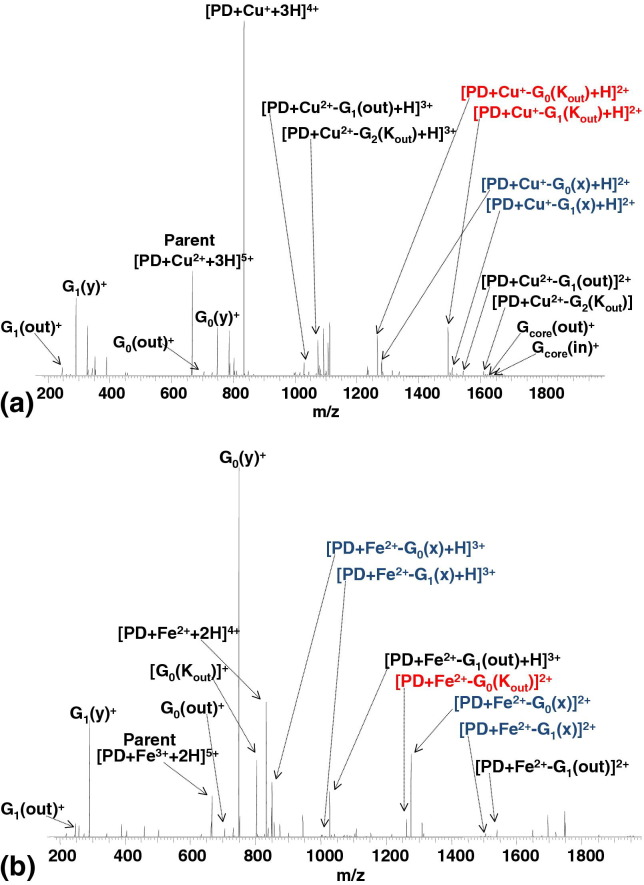
ECD FT-ICR mass spectrum of (**a**) [PD + Cu^2+^ + 3H]^5+^ ions and (**b**) [PD + Fe^3+^ + 2H]^5+^ ions. PD = PAMAM dendrimer. K cleavages are marked in red; *a/x* cleavages are marked in blue.

As described above, the ECD of protonated PAMAM dendrimers has been determined previously [21]. The dominant fragmentation channel for ECD of protonated PAMAM dendrimers is cleavage at the tertiary amines in the interior of the dendrimer. Pronounced amide bond cleavage in the interior of the dendrimer is also observed. Typically, the charge-reduced species [M + nH]^(n −1)+·^ constitutes a minor peak. The ECD MS/MS spectrum of [PD + Cu^2+^ + 3H]^5+^ is shown in Figure 1a and the fragments are detailed in Supplementary Table 1. The results suggest that electron capture occurs either by the metal ion or by the dendrimer ligand. The base peak in this mass spectrum can be assigned to the charge-reduced complex, [PD + Cu^+^ + 3H]^4+^, formed as a result of electron capture by the divalent copper ion. A series of peaks corresponding to fragment ions that arise from dissociation of this charge-reduced complex were also observed. For example, fragments formed as a result of cleavage within generation G_0_ of the dendrimer ligand, [PD + Cu^+^ − G_0_(K_out_) + H]^2+^ and [PD + Cu^+^ − G_0_(x) + H]^2+^, were found. Fragments containing Cu^+^ that resulted from cleavage in generation G_1_ were also apparent. No Cu^+^-containing fragments from generation G_2_ were observed. All of the Cu^+^-containing fragments derived from cleavages surface-side of the tertiary amines (K cleavages, marked red on the mass spectrum) or core-side of the amide (*a/x* cleavage, marked blue on the mass spectrum). Those cleavages are minor or non-existent in the ECD of protonated dendrimers. A series of fragment ions containing Cu^2+^, e.g., [PD + Cu^2+^ − G_1_(out)]^2+^ and [PD + Cu^2+^ − G_1_(out) + H]^3+^, was also observed. Presumably, those fragments were the result of electron capture by the dendrimer ligand. The presence of Cu^2+^-containing fragments resulting from cleavage surface-side of the tertiary amines (K cleavages), e.g., [PD + Cu^2+^ − G_2_(K_out_) + H]^3+^ and [PD + Cu^2+^ − G_2_(K_out_)]^2+^, demonstrates the influence of the metal ion on fragmentation behavior: those cleavages are rare in the ECD of protonated PAMAM dendrimer ions. In addition, nonmetal-containing fragments resulting from cleavage at tertiary amine or amide bonds were observed. It is not possible to say whether these fragments are the result of electron capture by the metal or ligand, however, as they reflect the patterns observed for protonated species, we speculate they arise following electron capture by the ligand. The following fragments fall within this category: G_1_(out)^+^, G_0_(out)^+^, G_core_(out)^+^, G_core_(in)^+^, G_1_(y)^+^ and G_0_(y)^+^. For comparison, the ECD mass spectrum of [PD + Cu^2+^ + 2H]^4+^ is shown in Supplemental Figure 1. Cu^+^-containing fragments deriving from cleavage surface-side of the tertiary amines (K cleavage) and core-side of the amide (*a/x* cleavage), and Cu^2+^-containing fragments deriving from K cleavage are observed.

The ECD MS/MS spectrum of [PD + Fe^3+^ + 2H]^5+^ complex is shown in Figure 1b. Fragments are detailed in Supplementary Table 1. Intense peaks corresponding to the charge-reduced species formed following metal ion electron capture, and fragments thereof, are observed. The Fe^2+^-containing fragments include [PD + Fe^2+^ − G_0_(x)]^2+^, [PD + Fe^2+^ − G_0_(K_out_)]^2+^, [PD + Fe^2+^ − G_1_(out)]^2+^, [PD + Fe^2+^ −G_1_(x)]^2+^, [PD + Fe^2+^ − G_0_(x) + H]^3+^, [PD + Fe^2+^ − G_1_(out) + H]^3+^, and [PD + Fe^2+^ − G_1_(x) + H]^3+^. No Fe^3+^-containing fragments were observed, suggesting that the competition between electron capture by the metal ion and the ligand is weighted in the former's favor. Again, cleavages core-side of the amide (*a/x*) are prevalent (marked blue on the mass spectrum). Cleavage surface-side of the tertiary amine (K) was also observed (marked red on the mass spectrum). Unlike the Cu^2+^ complex, the ECD mass spectrum of the Fe^3+^ complex is dominated by fragments arising from cleavage at amide bonds: G_0_(y)^+^ and G_1_(y)^+^, and at tertiary amines of dendrimer ligand: G_0_(out)^+^, G_1_(out)^+^, and G_0_(K_out_)^+^. It is not clear whether these fragments are the result of electron capture by the metal ion or the dendrimer ligand.

**Figure 2:**
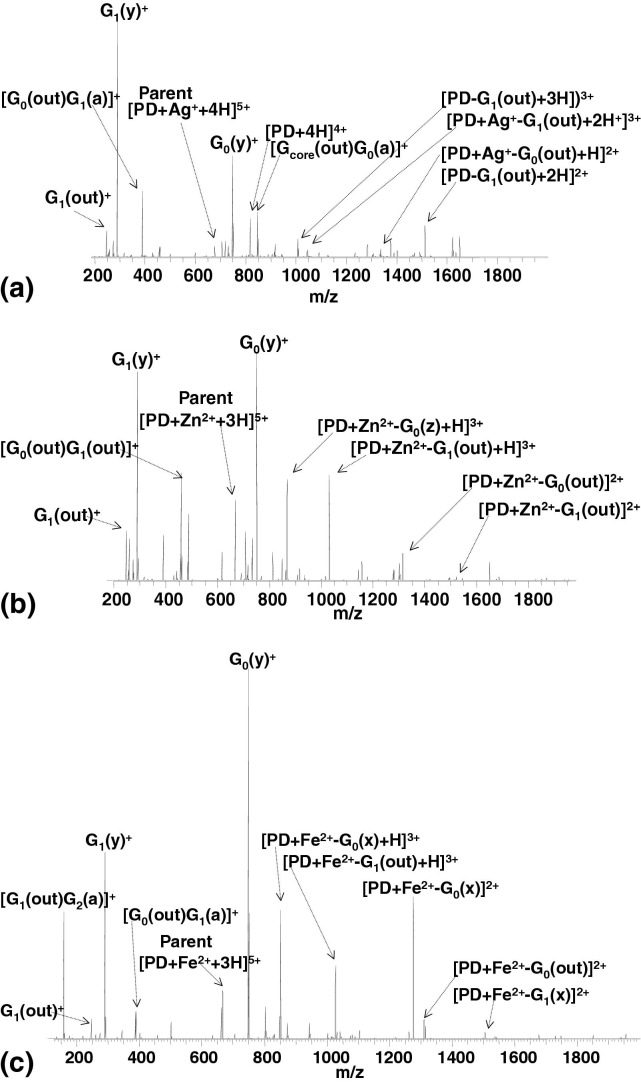
ECD FT-ICR mass spectrum of (**a**) [PD + Ag^+^ + 4H]^5+^ ions; (**b**) [PD + Zn^2+^ + 3H]^5+^ ions; (**c**) [PD + Fe^2+^ + 3H]^5+^ ions. PD = PAMAM dendrimer.

The ECD MS/MS spectra (see Figure 2) for complexes of Ag^+^, Zn^2+^, and Fe^2+^ with PAMAMG2OH dendrimer ligands are very similar. As seen for protonated PAMAMG2OH dendrimer ions [21], ECD of [PD + Ag^+^ + 4H]^5+^, [PD + Zn^2+^ + 3H]^5+^, and [PD + Fe^2+^ + 3H]^5+^ complexes are dominated by cleavage at the tertiary amines and amide bonds of PAMAMG2OH ligand. Typically, fragmentation occurs in the innermost generations of the dendrimer. The fragments are detailed in Supplementary Table 2. Electron capture by the metal ions in the Zn^2+^ and Fe^2+^ complexes was not observed. A peak corresponding to [PD + 4H]^4+^ was observed following ECD of [PD + Ag^+^ + 4H]^5+^. That species must be the result of electron capture by Ag^+^. Nevertheless, no dendrimer fragments containing Ag(0) were observed. For comparison, the ECD mass spectrum of [PD + Ag^+^ + 2H]^3+^ is shown in Supplemental Figure 2. Peaks corresponding to fragments deriving from cleavage at tertiary amines and amide bonds in the inner generations dominate the spectrum. A peak corresponding to [PD + 2H]^2+^ was also observed. No Ag(0)-containing dendrimer fragments were observed.

The results suggest that the ECD behavior of these complexes depends strongly on the properties of the metal ions. These findings are supported by previous work, which showed that the nature of the metal cation dictates the ECD of peptide [23] and lipid complexes [24, 25]. In the present case, the electron can be captured either by the metal ion or by the PAMAMG2OH ligand. When the electron is captured by the PAMAMG2OH ligand, the ECD is due to protonation of the tertiary amines and the presence of amide bonds in the dendrimer backbone and can be explained on the basis of a charge solvation model [20, 21]. When the electron is captured by the metal (Fe^3+^ and Cu^2+^), two main fragmentation channels are observed: *a/x* cleavage (core-side of the amide) and K cleavage (surface side of the tertiary amine). On the basis of electron paramagnetic resonance (EPR) experiments, Tomalia and coworkers [26] reported that in copper-dendrimer complexes the metal ion could be coordinated at various internal or external locations of the dendrimer. Crooks and coworkers [27], however, suggested that dendrimers with amidoethanol surface groups form metal ion complexes with interior tertiary amines. A study of Cu(II) complexes of a generation G_0_ PAMAM dendrimer by Nolan et al. [28] showed that for [PD + Cu^2+^ + 4H]^6+^, the metal was coordinated via the two core tertiary amines, and three amide oxygens (two equatorial and one axial). The complex [PD + Cu^2+^ + 2H]^4+^ was coordinated via the two core tertiary amines, one axial amide oxygen and two equatorial amide nitrogens. It is not clear which, if not both, coordination chemistries exist for the [PD + Cu^2+^ + 3H]^5+^ complex studied here. As Fe(III) also shows octahedral stereochemistry, it is reasonable to assume that [PD + Fe^3+^ + 2H]^5+^ is also coordinated via the two core tertiary amines, one axial amide oxygen and either two amide nitrogens or two further amide oxygens. (Further evidence for this stereochemistry derives from the CID data discussed below). For both the Fe^3+^ and Cu^2+^ complexes, the pronounced *a/x* cleavage can be explained by coordination of the metal by amide nitrogens and/or oxygens. Although the metal-containing fragments reveal *a/x* cleavage, complementary fragments are not observed. Rather, nonmetal-containing *y* fragments are prevalent. Those fragments may arise from capture of the electron by the ligand (see below) or they may be the result of secondary cleavage of *x* ions via loss of CO. In addition to the coordination chemistry described above, we hypothesize that for larger PAMAM dendrimers, the outer tertiary amines (G_0_, G_1_, G_2_) may be involved in metal complexation, replacing the amide nitrogens/oxygens. That stereochemistry may explain the pronounced K cleavage. No fragments were observed that contained the dendrimer core but not the metal ion, suggesting that the core is essential in coordination of the metal.

In addition to the metal ion, the complexes studied also varied in the number of protons attached: The Fe^3+^ complex has two protons, the Fe^2+^, Zn^2+^, and Cu^2+^ complexes have three protons and the Ag^+^ complex has four protons. We have observed previously that for the PAMAMG2OH dendrimer ECD behavior is independent of the number of protons attached [21]. That finding was echoed by Oh and coworkers [20] in their work on the ECD of PAMAMG3OH. Their results showed that dominant cleavage at the tertiary amines and production of y fragments was observed consistently over all charge states studied (4+ through 8+). The results here suggest that ECD of metal complexes of PAMAMG2OH is independent of the number of the protons attached: ECD of [PD + Ag^+^ + 2H]^3+^ (Supplemental Figure 2) reflects that of [PD + Ag^+^ + 4H]^5+^ and not that of [PD + Fe^3+^ + 2H]^5+^. The ECD of [PD + Cu^2+^ + 2H]^4+^ (Supplemental Figure 1) reflects that of [PD + Cu^2+^ + 3H]^5+^. The ECD of [PD + Cu^2+^ + 3H]^5+^ (Figure 1a) does not mirror that of [PD + Zn^2+^ + 3H]^5+^ and [PD + Fe^2+^ + 3H]^5+^ (Figure 2b and c).

A study on the ECD of complexes of divalent metal ions and diacylglycerophosphocholine by O'Hair and coworkers [24] revealed that those metals with the highest second ionization energies stabilized the charge-reduced complexes via change in the oxidation state, whereas those metals with lowest second ionization energies promoted electron capture by (and fragmentation of) the ligand. We have found that, for those metals with 3d valence electrons, the ECD behavior of metal ion–PAMAM complexes also tracks with the highest ionization energy (IE): The Fe^3+^ (IE3 30.6 eV) and Cu^2+^ (IE2 20.3 eV) stabilize the charge-reduced complexes, via change in the oxidation state of the metal ion. Detection of fragments containing Fe^2+^ and Cu^+^ confirms electron capture by the metal ions. (In fact, no Fe^3+^-containing fragments were observed). For metal ions with lower ionization energies, Zn^2+^ (IE2 17.9 eV), and Fe^2+^ (IE2 16.1 eV), formation of charge-reduced complexes and change in oxidation state of metal ion is not observed. Instead, electron capture by, and fragmentation of, the PAMAMG2OH dendrimer ligand is promoted. For the Ag^+^ complexes (IE1 7.6 eV; 4d valence electrons), the presence of [PD + nH]^*n*+^species suggests that electron capture by the metal ion does occur. However, no Ag(0)-containing dendrimer fragments are observed.

### Collision-Induced Dissociation of Complexes of PAMAM Dendrimer with Metal ions: Ag^+^, Cu^2+^, Zn^2+^, Fe^2+^, and Fe^3+^

**Figure 3:**
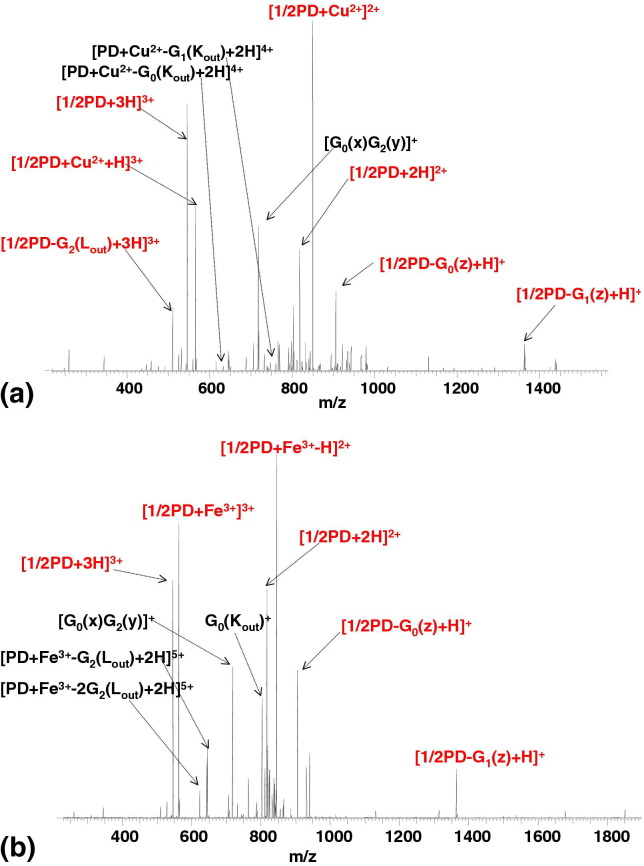
CID FT-ICR mass spectrum of (**a**) [PD + Cu^2+^ + 3H]^5+^ ions; (**b**) [PD + Fe^3+^ + 2H]^5+^ ions. PD = PAMAM dendrimer. Cleavages between carbon atoms in the ethylenediamine core are marked in red.

The CID mass spectrum of the [PD + Cu^2+^ + 3H]^5+^ complex, shown in Figure 3a, reveals four intense signals, which can be assigned to [1/2PD + Cu^2+^]^2+^, [1/2PD + Cu^2+^ + H]^3+^, [1/2PD + 2H]^2+^, and [1/2PD + 3H]^3+^, respectively (fragments are detailed in Supplementary Table 3). These fragment ions are the result of cleavage between the two carbon atoms of the ethylenediamine core of the dendrimer ligand. Other abundant fragments, including [1/2PD − G_2_(L_out_) + 3H]^3+^, [1/2PD − G_0_(z) + H]^+^ and [1/2PD − G_1_(z) + H]^+^ also involve cleavage in the core of dendrimer (marked red on the mass spectrum). (For comparison, Supplemental Figure 3 shows the CID mass spectrum of [PD + Cu^2+^ + 2H]^4+^. Similar fragmentation behavior was observed). That fragmentation behavior is particularly surprising considering the CID of protonated PAMAMG2OH dendrimers [19, 21], in which L and K cleavages, and combinations of the two cleavage types in various stoichiometries, from the outermost generation were the dominant processes. No core C–C bond cleavage was observed in the CID of protonated PAMAM precursor ions. The results suggest that the CID of the [PD + Cu^2+^ + 3H]^5+^ complex is linked with the coordination of the divalent copper ion to the PAMAMG2OH dendrimer ligand. As described above, it has been suggested that coordination of the Cu^2+^ ion involves the tertiary amines of the dendrimer core. Further evidence for that was provided by the ECD data obtained here. Two salient points arise from the CID data. Firstly, the Cu^2+^ ion is attached close to dendrimer core. That idea is supported by the fact that the Cu^2+^-containing fragment [PD + Cu^2+^ − G_0_(K_out_) + 2H]^4+^, which arises through the loss of G_0_(K_out_) from the innermost generation of the complex, is observed, but Cu^2+^-containing fragments from the outer generations, e.g., [Cu^2+^+ G_2_(K_out_) + nH]^(*n* + 2^)^+^ or [Cu^2+^ +G_1_(K_out_) + nH]^(*n* + 2^)^+^, are not. Secondly, the abundance of fragments resulting from the cleavage of the core C–C bond suggests that both core tertiary amines coordinate the metal ion, and as a result the C–C bond is weakened.

The CID MS/MS spectrum of [PD + Fe^3+^ + 2H]^5+^ precursor ions is shown in Figure 3b. Fragments are detailed in Supplementary Table 3. The most abundant peaks correspond to [1/2PD + Fe^3+^ − H]^2+^, [1/2PD + Fe^3+^]^3+^, [1/2PD + 3H]^3+^, and [1/2PD + 2H]^2+^, respectively, and are formed as a result of the cleavage between carbon atoms in the ethylenediamine core (marked red on the mass spectrum). The presence of metal-containing fragments such as [PD + Fe^3+^ − G_2_(L_out_) + 2H]^5+^ suggest that the metal ion is coordinated to the interior of the dendrimer. It was postulated earlier that coordination of Fe(III) also involves the core tertiary amines. We hypothesize that coordination of the Fe^3+^ ion weakens the core C–C bond thus promoting its cleavage following vibrational excitation.

**Figure 4:**
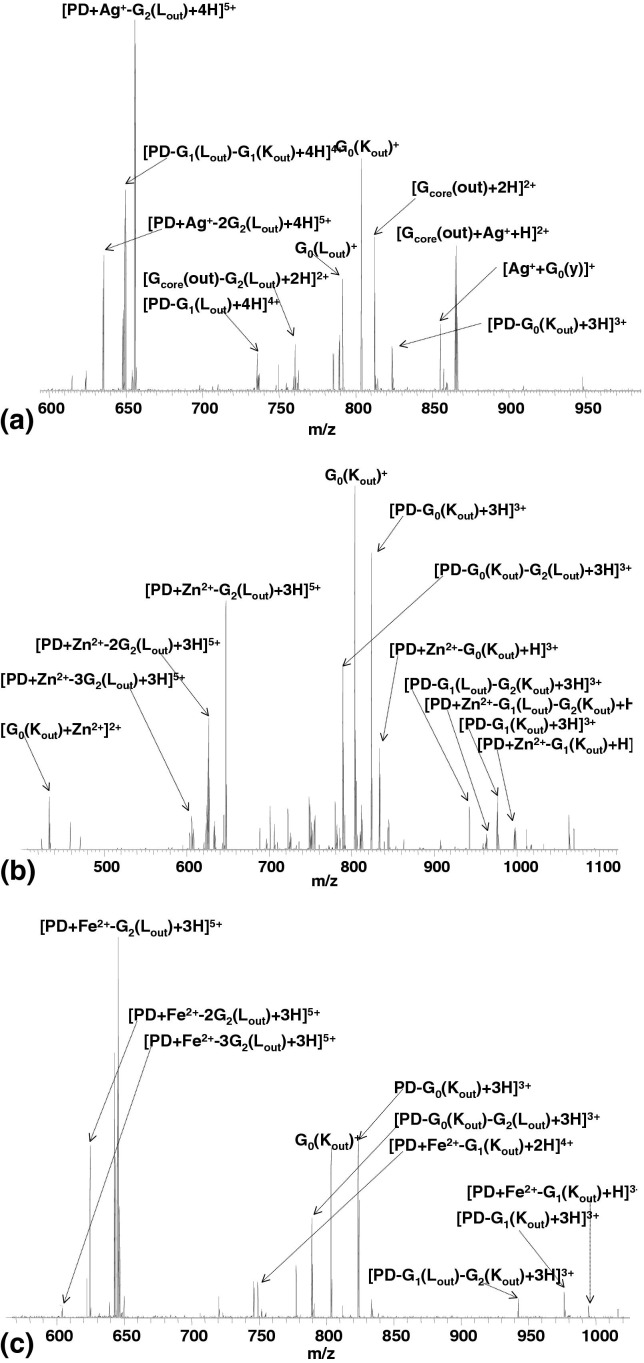
CID FT-ICR mass spectrum of (**a**) [PD + Ag^+^ + 4H]^5+^ ions; (**b**) [PD + Zn^2+^ + 3H]^5+^ ions; (**c**) [PD + Fe^2^ + 3H]^5+^ ions. PD = PAMAM dendrimer.

The CID mass spectrum of the [PD + Ag^+^ + 4H]^5+^complex, shown in Figure 4a, is dominated by peaks corresponding to fragments arising from L and K cleavages in the outer G_1_ and G_2_ generations (i.e., [PD + Ag^+^ − G_2_(L_out_) + 4H]^5+^, [PD + Ag^+^ − 2G_2_(Lout) + 4H]^5+^, and [PD − G_1_(L_out_) − G_1_(K_out_) + 4H]^4+^). In addition, there are abundant peaks corresponding to [G_core_(out) + 2H]^2+^, [G_core_(out) + Ag^+^+ H]^2+^ and G_0_(K_out_)^+^. No cleavage of the core C–C bond was observed. The fragments are detailed in Supplementary Table 4. Most of the fragments observed for the [PD + Ag^+^+ 4H]^5+^complex were also observed for the protonated [PD + 6H]^6+^ ions in our earlier study [21]. (Similar fragmentation was recorded following CID of [PD + Ag^+^ + 2H]^3+^, see Supplemental Figure 4). In all cases, the most intense signals correspond to loss of G_2_(L_out_) fragments. Similar fragmentation behavior has been reported for doubly charged [PD + Ag^+^ +H]^2+^ ions [19]. Thus, apparently, the CID of silver ion/PAMAMG2OH dendrimer complexes does not depend on the number of mobile protons. The presence of the silver-containing fragments [G_core_(out) + Ag^+^ + H]^2+^ and [Ag^+^ + G_0_(y)]^+^, and absence of silver-containing fragments from generations G_2_ or G_1_, suggest that the silver ion is bound close to the core of the dendrimer. However, CID of [PD + Ag + 4H]^5+^ does not result in any fragments deriving from cleavage of the core C–C bond. According to our hypothesis that observation suggests that coordination of the Ag^+^ ion does not involve both core tertiary amines. That idea is further corroborated by the presence in the mass spectrum of [G_core_(out) + 2H]^2+^ and [G_core_(out) + Ag + H]^2+^. In their study of Ag(I) complexes of a generation G_0_ PAMAM dendrimer, Nolan et al. [28] found two major pH-dependent species. The first species was [PD + Ag + 3H]^4+^ and the second was [PD + 2Ag]^2+^. The structure proposed for the mononuclear complex involved trigonal planar coordination via the two core tertiary amines and an amide nitrogen; and for the dinuclear complex, linear coordination of one of the core tertiary amines and a second, outer, tertiary amine, possibly accompanied by additional interaction of an amide oxygen. The CID results for [PD + Ag + 4H]^5+^ suggest that of the two possibilities, the latter coordination structure is more likely.

The CID behavior of [PD + Zn^2+^+ 3H]^5+^ complex, shown in Figure 4b, also reflects that observed for the protonated dendrimer [21]. The majority of the fragments derive from L and K cleavages in generations G_2_, G_1_, and G_0_. The fragments observed are detailed in Supplementary Table 4. Peaks corresponding to [PD − G_0_(K_out_) + 3H]^3+^, [PD − G_0_(K_out_) − G_2_(L_out_) + 3H]^3+^, [G_0_(K_out_) + Zn^2+^]^2+^, and the lack of Zn-containing core fragments suggest that the Zn is either not, or weakly, bound to the core of the dendrimer. Exclusive loss of the zinc ion from the [PD + Zn^2+^ + 3H]^5+^complex is not observed. Fragments deriving from cleavage in generations G_0_ and G_1_ are observed both with and without the metal ion, e.g., [PD − G_1_(L_out_) − G_2_(K_out_) + H]^3+^ and [PD + Zn^2+^ − G_1_(L_out_) − G_2_(K_out_) + H]^3+^; G_0_(K_out_)^+^ and [G_0_(K_out_) + Zn^2+^]^2+^; [PD − G_0_(K_out_) + 3H]^3+^ and [PD + Zn^2+^ − G_0_(K_out_) + H]^3+^. The same is not true for fragments originating from cleavage in the outermost generation (G_2_): the fragments [PD + Zn^2^ − G_2_(L_out_) + 3H]^5+^ and [PD + Zn^2+^ − 2G_2_(L_out_) + 3H]^5+^ are not accompanied by nonmetal-containing partners, suggesting the metal ion is not bound to G_2_.

Similar overall CID behavior was observed for the [PD + Fe^2+^ + 3H]^5+^ complex (Figure 4c, Supplementary Table 4). The dominant pathways involved K and L cleavages in the outermost generation (G_2_): ([PD + Fe^2+^ − G_2_(L_out_) + 3H]^5+^ and [PD + Fe^2+^ − 2G_2_(L_out_) + 3H]^5+^ are formed. The Fe^2+^ does not appear to be coordinated to G_2_. Fragments from G_1_ are observed both with and without Fe^2+^, e.g., [PD − G_1_(K_out_) + 3H]^3+^, and [PD + Fe^2+^ − G_1_(K_out_) + H]^3+^. The stereochemistry of Fe^2+^ complexes is typically octahedral or tetrahedral. It is not clear which is true in the present example, however the results suggest that the Fe^2+^ is at best weakly bound to only one of the core tertiary amines. Note the marked difference between the CID of the Fe^2+^ complex and the Fe^3+^ complex (Figure 3b).

## Conclusions

We have investigated the electron capture dissociation (ECD) and collision-induced dissociation (CID) of complexes of the PAMAMG2OH dendrimer with different metal ions (Ag^+^, Cu^2+^, Zn^2+^, Fe^2+^, and Fe^3+^). Complementary information regarding the site and coordination of the metal ions can be obtained from the two techniques. For the Cu^2+^ complex and the Fe^3+^ complex, electron capture by the metal resulted in pronounced *a/x* cleavage suggesting that amide nitrogen atoms and/or oxygen atoms were involved in metal coordination. In addition, the pronounced K cleavage suggested that outer tertiary amines were also involved in metal coordination. The CID of these two complexes indicates that both the core tertiary amines are involved in metal coordination. The ECD of the Zn^2+^ and Fe^2+^ complexes mimicked that of protonated PAMAM dendrimer ions, i.e., capture of the electron by the ligand was observed. Capture of the electron by the Ag^+^ ion resulted in loss of the metal. Fragments observed following ECD of the Ag^+^ complexes were the result of electron capture by the ligand. For the fourth period metals (3d valence shells), the ECD behavior of the metal complexes tracked with the highest ionization energy: Electron capture by the metal ion was observed for those with the highest ionization energy (Fe^3+^ and Cu^2+^) and by the ligand for those with the lowest (Zn^2+^ and Fe^2+^). The CID behavior of the Ag^+^ complex suggests that the metal is coordinated by one, but not both, of the core tertiary amines, while the CID of the Zn^2+^ and Fe^2+^ suggests that the metal is located in the interior of the dendrimer (G_0_, G_1_) but is not necessarily coordinated to the core.

## Supplementary data

Supplemental Figure 1 ECD FT-ICR mass spectrum of [PD + Cu^2+^ + 2H]^4+^ ions. PD = PAMAM dendrimer. K cleavages are marked in red; *a/x* cleavages are marked in blue.

Supplemental Figure 2 ECD FT-ICR mass spectrum of [PD + Ag^+^ + 2H]^3+^ ions. PD = PAMAM dendrimer.

Supplemental Figure 3 CID FT-ICR mass spectrum of [PD + Cu^2+^ + 2H]^4+^ ions. PD = PAMAM dendrimer. Cleavages between carbon atoms in the ethylenediamine core are marked in red on the spectrum.

Supplemental Figure 4 CID FT-ICR mass spectrum of [PD + Ag^+^ + 2H]^3+^ ions. PD = PAMAM dendrimer.
