# Supplementary figures and images for: Electron Capture Dissociation and Collision-Induced Dissociation of Metal Ion (Ag+, Cu2+, Zn2+, Fe2+, and Fe3+) Complexes of Polyamidoamine (PAMAM) Dendrimers
Source: J Am Soc Mass Spectrom. 2009 Apr;20(4):674–81. doi: 10.1016/j.jasms.2008.12.013 (PMC2667233; doi:10.1016/j.jasms.2008.12.013)

# Suppl. Figure 1

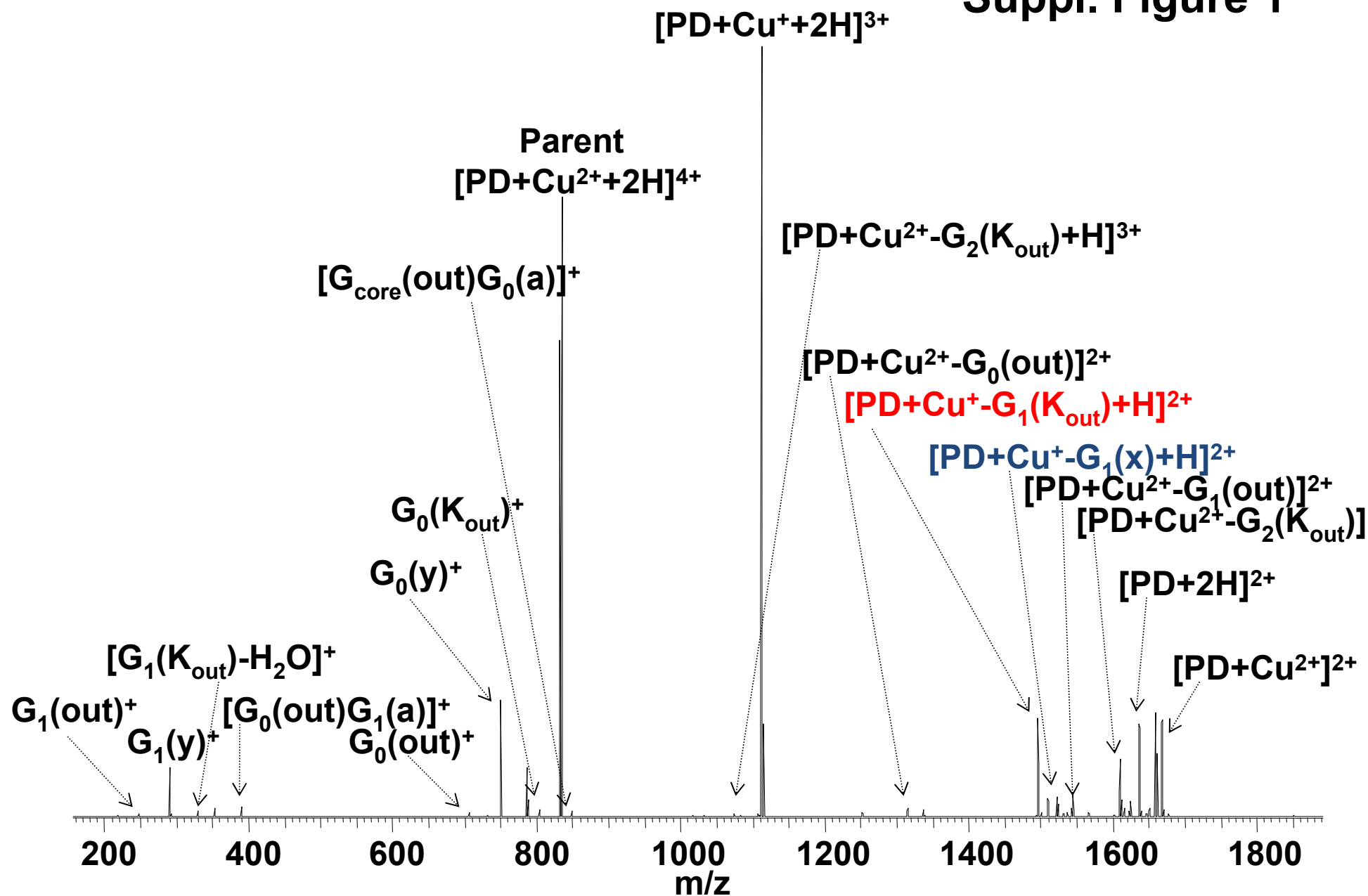

Supplement: Supplemental Figure 1 — ECD FT-ICR mass spectrum of [PD + Cu2+ + 2H]4+ ions. PD = PAMAM dendrimer. K cleavages are marked in red; a/x cleavages are marked in blue. [file mmc1.pdf]

# Suppl. Figure 2

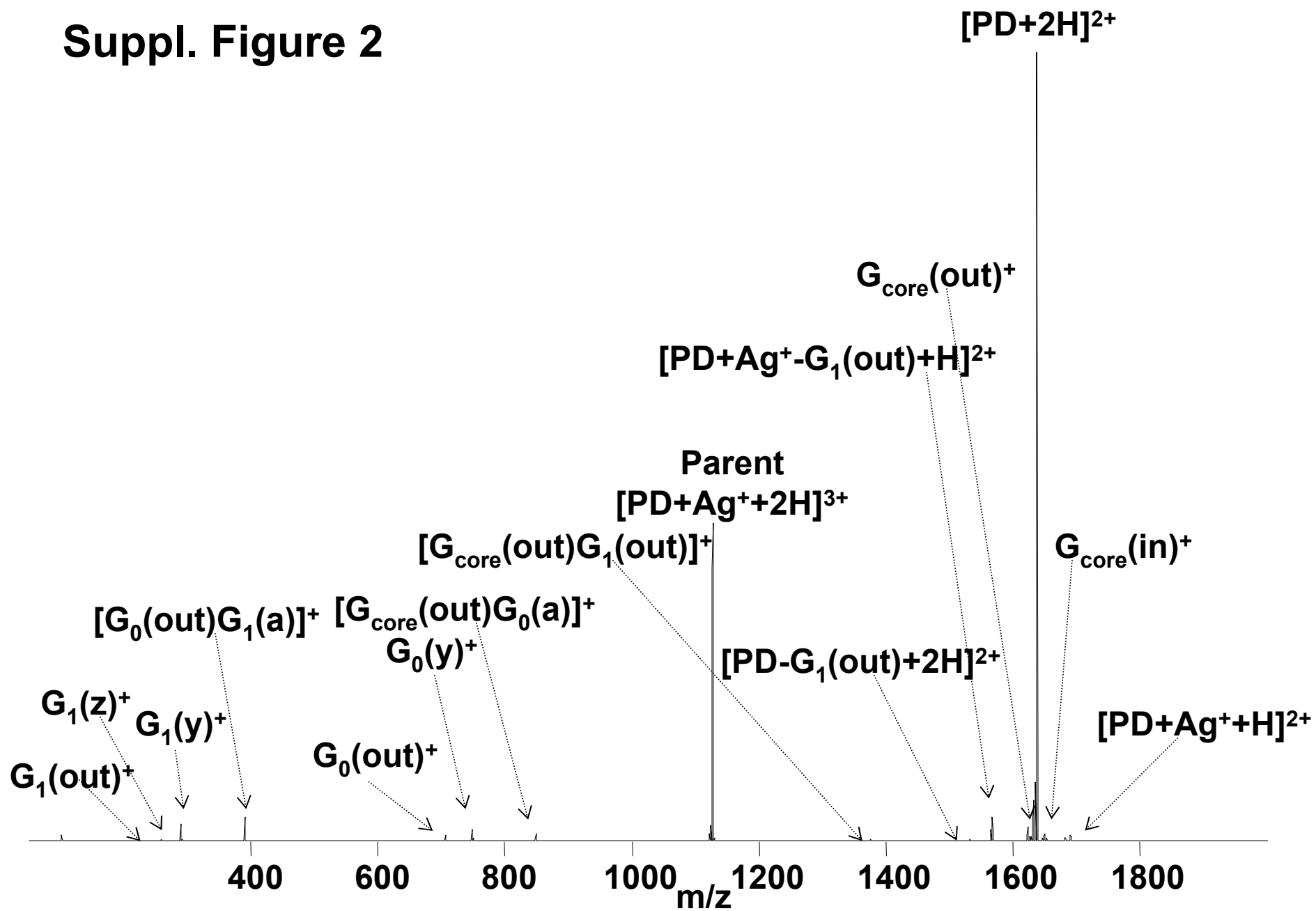

Supplement: Supplemental Figure 2 — ECD FT-ICR mass spectrum of [PD + Ag+ + 2H]3+ ions. PD = PAMAM dendrimer. [file mmc2.pdf]

Suppl. Figure 3

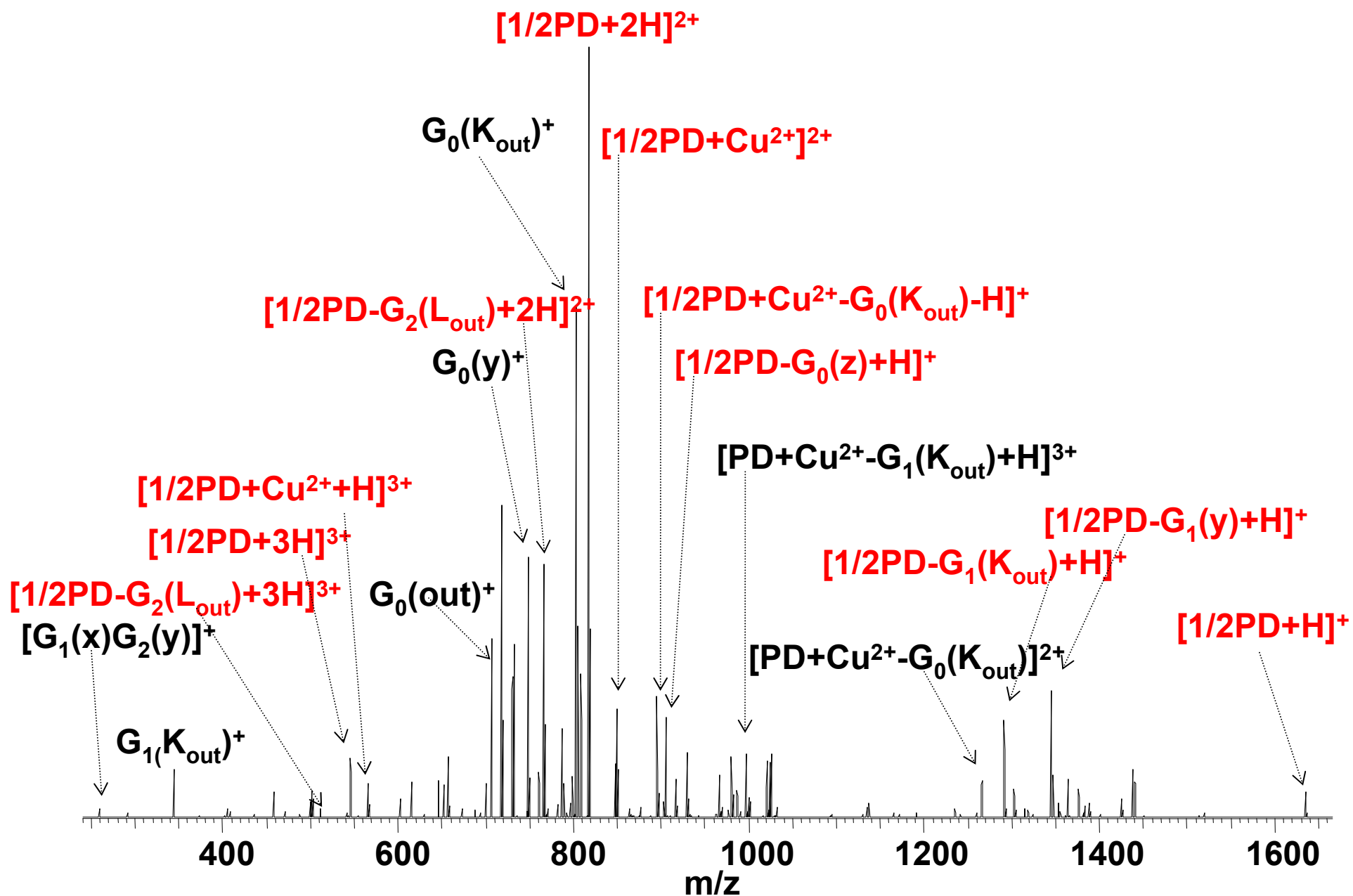

Supplement: Supplemental Figure 3 — CID FT-ICR mass spectrum of [PD + Cu2+ + 2H]4+ ions. PD = PAMAM dendrimer. Cleavages between carbon atoms in the ethylenediamine core are marked in red on the spectrum. [file mmc3.pdf]

Suppl. Figure 4

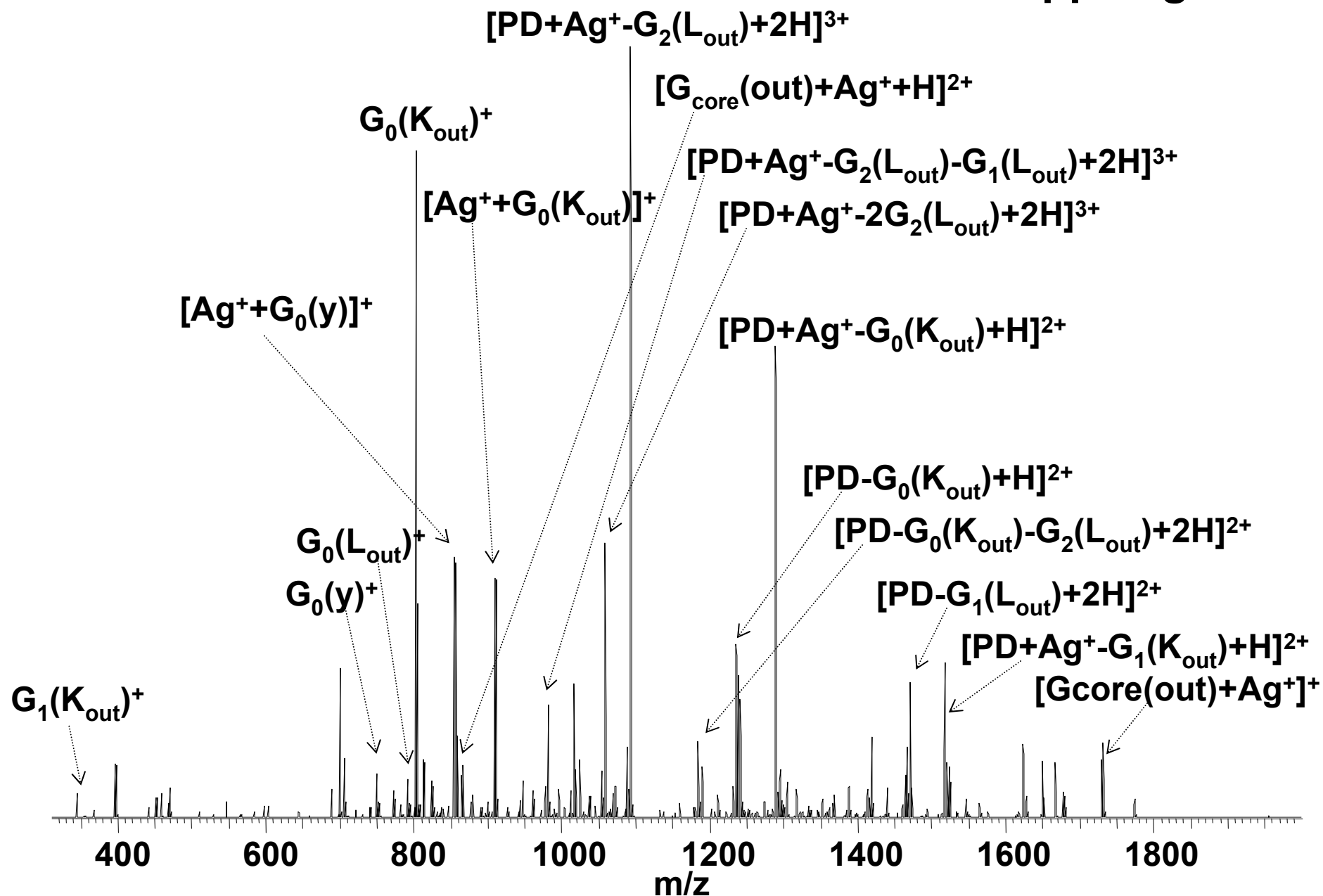

Supplement: Supplemental Figure 4 — CID FT-ICR mass spectrum of [PD + Ag+ + 2H]3+ ions. PD = PAMAM dendrimer. [file mmc4.pdf]
