# Supplementary material for: Electron Capture Dissociation and Collision-Induced Dissociation of Metal Ion (Ag+, Cu2+, Zn2+, Fe2+, and Fe3+) Complexes of Polyamidoamine (PAMAM) Dendrimers
Source: J Am Soc Mass Spectrom. 2009 Apr;20(4):674–81. doi: 10.1016/j.jasms.2008.12.013 (PMC2667233; doi:10.1016/j.jasms.2008.12.013)
Supplement: Supplementary Table 1 [file mmc5.pdf]

Supplemental Table 1.The most abundant fragment ions observed following ECD of [a]$[PD+Cu^{2+}+3H]^{5+}$, and [b]$[PD+Fe^{3+}+2H]^{5+}$ ions, PD= PAMAMG2OH.

m/z measured	*m/z* calculated	Assignment
[a]248.1604, [b]248.1602	248.1610	$G_1(out)^+$
[b]260.1603	260.1605	$[G_1(x)G_2(y)]^+$
[a]291.2026, [b]291.2024	291.2027	$G_1(y)^+$
[a]345.2131, [b]345.2127	345.2138	$G_1(K_{out})^+$
[a]329.2182	329.2189	$[G_1(K_{out})-H_2O]^+$
[a]389.2635, [b]389.2627	389.2633	$[G_0(out)G_1(a)]^+$
[b]459.2918	459.2926	$[G_0(out)G_1(out)]^+$
[a]706.4452, [b]706.4441	706.4458	$G_0(out)^+$
[a]732.4612, [b]732.4600	732.4614	$G_0(z)^+$
[a]749.4875, [b]749.4863	749.4880	$G_0(y)^+$
[a]803.4981, [b]803.4967	803.4985	$G_0(K_{out})^+$
[b]831.9933	831.9958	$[PD+Fe^{2+}+2H]^{4+}$
[a]833.9954	833.9988	$[PD+Cu^{+}+3H]^{4+}$
[b]850.5003	850.5029	$[PD+Fe^{2+}-G_0(x)+H]^{3+}$
[a]996.5874	996.5913	$[PD+Cu+-G_1(K_{out})+2H]^{3+}$
[b]1003.2587	1003.2644	$[PD+Fe^{2+}-G_1(x)+H]^{3+}$
[b]1026.6039	1026.6075	$[PD+Fe^{2+}-G_1(out)+H]^{3+}$
[a]1028.9376	1028.9426	$[PD+Cu^{2+}-G_1(out)+H]^{3+}$
[a]1072.9678	1072.9725	$[PD+Cu^{2+}-G_2(K_{out})+H]^{3+}$
[a]1091.0176	1091.0185	$[PD+3H]^{3+}$
[a]1111.3223	1111.3270	$[PD+Cu^{2+}+H]^{3+}$
[a]1234.7770	1234.7785	$[PD-G_0(K_{out})+2H]^{2+}$
[b]1261.2291	1261.2350	$[PD+Fe^{2+}-G_0(K_{out})]^{2+}$
[a]1265.2340	1265.2411	$[PD+Cu^{+}-G_0(K_{out})+H]^{2+}$

[b]1275.2447	1275.2505	$[PD+Fe^{2+}-G_0(x)]^{2+}$
[a]1279.2493	1279.2567	$[PD+Cu^{+}-G_0(x)+H]^{2+}$
[b]1309.7560	1309.7614	$[PD+Fe^{2+}-G_0(out)]^{2+}$
[a]1494.3766	1494.3834	$[PD+Cu^{+}-G_1(K_{out})+H]^{2+}$
[b]1504.4050	1504.3930	$[PD+Fe^{2+}-G_1(x)]^{2+}$
[a]1508.3915	1508.3988	$[PD+Cu^{+}-G_1(x)+H]^{2+}$
[a]1522.3918	1522.3962	$[PD+Cu^{+}-G_1(y)+H]^{2+}$
[b]1539.3983	1539.4077	$[PD+Fe^{2}-G_1(out)]^{2+}$
[a]1542.9039	1542.9100	$[PD+Cu^{2+}-G_1(out)]^{2+}$
[a]1609.4513	1609.4586	$[PD+Cu^{2+}-G_2(K_{out})]^{2+}$
[a]1623.0138	1623.0163	$G_{core}(out)^{+}$
[a]1649.0307, [b]1649.0253	1649.0320	$G_{core}(in)^{+}$
