# Supplementary material for: Electron Capture Dissociation and Collision-Induced Dissociation of Metal Ion (Ag+, Cu2+, Zn2+, Fe2+, and Fe3+) Complexes of Polyamidoamine (PAMAM) Dendrimers
Source: J Am Soc Mass Spectrom. 2009 Apr;20(4):674–81. doi: 10.1016/j.jasms.2008.12.013 (PMC2667233; doi:10.1016/j.jasms.2008.12.013)
Supplement: Supplementary Table 2 [file mmc6.pdf]

**Supplemental Table 2**.The most abundant fragment ions observed following ECD of [c][PD+Ag$^{+}$+4H]$^{5+}$, [d][PD+Zn$^{2+}$+3H]$^{5+}$, [e][PD+Fe$^{2+}$+3H]$^{5+}$ ions. PD= PAMAMG2OH.

| *m/z* measured | *m/z* calculated | Assignment |
|---|---|---|
| [e]160.1207 | 160.1206 | $[G_1(out)G_2(a)]^+$ |
| [c]248.1605, [d]248.1605, [e]248.1602 | 248.1610 | $G_1(out)^+$ |
| [d]260.1604 | 260.1605 | $[G_1(x)G_2(y)]^+$ |
| [c]274.1762, [d]274.1761, [e]274.1758 | 274.1761 | $G_1(z)^+$ |
| [c]291.2027, [d]291.2027, [e]291.2023 | 291.2027 | $G_1(y)^+$ |
| [c]345.2133, [d]345.2128, [e]345.2129 | 345.2138 | $G_1(K_{out})^+$ |
| [c]389.2633, [d]389.2634, [e]389.2627 | 389.2633 | $[G_0(out)G_1(a)]^+$ |
| [c]459.2927, [d]459.2928, [e]459.2911 | 459.2926 | $[G_0(out)G_1(out)]^+$ |
| [c]706.4463, [d]706.4465, [e]706.4443 | 706.4458 | $G_0(out)^+$ |
| [c]718.4464 | 718.4458 | $[G_0(x)G_2(y)]^+$ |
| [c]732.4620, [d]732.4628 | 732.4614 | $G_0(z)^+$ |
| [c]749.4886, [d]749.4889, [e]749.4861 | 749.4880 | $G_0(y)^+$ |
| [c]803.4988, [d]803.4986, [e]803.4960 | 803.4985 | $G_0(K_{out})^+$ |
| [c]818.5161 | 818.5157 | $[PD+4H]^{4+}$ |
| [e]831.9924 | 831.9958 | $[PD+Fe^{2+}+2H]^{4+}$ |
| [c]848.5571, [d]848.5576, [e]848.5537 | 848.5564 | $[G_{core}(out)G_0(a)]^+$ |
| [e]850.5008 | 850.5029 | $[PD+Fe^{2+}-G_0(x)+H]^{3+}$ |
| [d]867.8395 | 867.8387 | $[PD+Zn^{2+}-G_0(z)+H]^{3+}$ |
| [c]917.5789 | 917.5779 | $[G_{core}(out)G_0(out)]^+$ |
| [c]1008.6334 | 1008.6343 | $[PD-G_1(out)+3H]^{3+}$ |
| [e]1026.6028 | 1026.6075 | $[PD+Fe^{2+}-G_1(out)+H]^{3+}$ |
| [d]1029.2738 | 1029.2724 | $[PD+Zn^{2+}-G_1(out)+H]^{3+}$ |
| [c]1043.9342 | 1043.9336 | $[PD+Ag^{+}-G_1(out)+2H]^{3+}$ |
| [e]1261.2319 | 1261.2350 | $[PD+Fe^{2+}-G_0(K_{out})]^{2+}$ |

| | | |
|---|---|---|
| [e]1275.2469 | 1275.2505 | $[PD+Fe^{2+}-G_0(x)]^{2+}$ |
| [c]1283.3075 | 1283.3049 | $[PD-G_0(out)+2H]^{2+}$ |
| [d]1301.2564 | 1301.2544 | $[PD+Zn^{2+}-G_0(z)]^{2+}$ |
| [e]1309.7560 | 1309.7614 | $[PD+Fe^{2+}-G_0(out)]^{2+}$ |
| [d]1314.2654 | 1314.2622 | $[PD+Zn^{2+}-G_0(out)]^{2+}$ |
| [c]1336.2553 | 1336.2540 | $[PD+Ag^{+}-G_0(out)+H]^{2+}$ |
| [c]1512.4498 | 1512.4478 | $[PD-G_1(out)+2H]^{2+}$ |
| [d]1521.3803 | 1521.3850 | $[PD+Zn^{2+}-G_1(y)]^{2+}$ |
| [c]1623.0185 | 1623.0163 | $G_{core}(out)^{+}$ |
| [c]1649.0345, [d]1649.0372 | 1649.0320 | $G_{core}\ (in)^{+}$ |
