# Supplementary material for: Electron Capture Dissociation and Collision-Induced Dissociation of Metal Ion (Ag+, Cu2+, Zn2+, Fe2+, and Fe3+) Complexes of Polyamidoamine (PAMAM) Dendrimers
Source: J Am Soc Mass Spectrom. 2009 Apr;20(4):674–81. doi: 10.1016/j.jasms.2008.12.013 (PMC2667233; doi:10.1016/j.jasms.2008.12.013)
Supplement: Supplementary Table 3 [file mmc7.pdf]

Supplemental Table 3.The most abundant fragment ions observed following CID of [f][$PD+Cu^{2+}+3H]^{5+}$ and [g][$PD+Fe^{3+}+2H]^{5+}$ ions, PD= PAMAMG2OH.

m/z measured	*m/z* calculated	Assignment
[f]248.1602	248.1610	$G_1(out)^+$
[f]260.1601, [g]260.1599	260.1605	$[G_1(x)G_2(y)]^+$
[f]345.2126, [g]345.2123	345.2138	$G_1(K_{out})^+$
[f]459.2913	459.2926	$[G_0(out)G_1(out)]^+$
[f]511.3210	511.3127	$[1/2PD-G_2(L_{out})+3H]^{3+}$
[f]525.3244	525.3264	$[1/2PD-G_2(y)+3H]^{3+}$
[f]545.6750, [g]545.6771	545.6771	$1/2PD+3H]^{3+}$
[f]560.3123	560.3177	$[1/2PD+Cu^{2+}+H-H_2O]^{3+}$
[g]563.6474	563.6506	$[1/2PD+Fe^{3+}]^{3+}$
[f]566.3155	566.3214	$[1/2PD+Cu^{2+}+H]^{3+}$
[g]624.5691	624.5727	$[PD+Fe^{3+}-2G_2(L_{out})+2H]^{5+}$
[f]633.1186	633.1244	$[PD+Cu^{2+}-G_0(K_{out})+2H]^{4+}$
[g]644.9820	644.9841	$[PD+Fe^{3+}-G_2(L_{out})+2H]^{5+}$
[f]646.5818	646.5863	$[PD+Cu^{2+}-G_2(L_{out})+3H]^{5+}$
[f]706.4428, [g]706.4415	706.4458	$G_0(out)^+$
[f]718.4427, [g]718.4415	718.4458	$[G_0(x)G_2(y)]^+$
[f]747.6888	747.6953	$[PD+Cu^{2+}-G_1(K_{out})+2H]^{4+}$
[f]803.4947, [g]803.4933	803.4985	$G_0(K_{out})^+$
[f]818.0078, [g]818.0087	818.0087	$[1/2PD+2H]^{2+}$
[g]844.9644	844.9720	$[1/2PD+Fe^{3+}-H]^{2+}$
[f]849.4753	849.4821	$[1/2PD+Cu^{2+}]^{2+}$
[f]905.5734, [g]905.5711	905.5784	$[1/2PD-G_0(z)+H]^+$
[f]1363.8495, [g]1363.8446	1363.8636	$[1/2PD-G_1(z)+H]^+$
