# Supplementary material for: Electron Capture Dissociation and Collision-Induced Dissociation of Metal Ion (Ag+, Cu2+, Zn2+, Fe2+, and Fe3+) Complexes of Polyamidoamine (PAMAM) Dendrimers
Source: J Am Soc Mass Spectrom. 2009 Apr;20(4):674–81. doi: 10.1016/j.jasms.2008.12.013 (PMC2667233; doi:10.1016/j.jasms.2008.12.013)
Supplement: Supplementary Table 4 [file mmc8.pdf]

Supplemental Table 4.The most abundant fragment ions observed following CID of [h]$[PD+Ag^{+}+4H]^{5+}$, [i]$[PD+Zn^{2+}+3H]^{5+}$, [j]$[PD+Fe^{2+}+3H]^{5+}$ ions. PD= PAMAMG2OH.

m/z measured	*m/z* calculated	Assignment
[i]248.1601	248.1610	$G_1(out)^{+}$
[h]345.2119, [i]345.2125, [j]345.2124	345.2138	$G_1(K_{out})^{+}$
[i]433.2084	433.2101	$[G_0(K_{out})+Zn^{2+}]^{2+}$
[i]459.2912	459.2926	$[G_0(out)G_1(out)]^{+}$
[j]603.9568	603.9601	$[PD+Fe^{2+}-3G_2(L_{out})+3H]^{5+}$
[j]624.5692	624.5727	$[PD+Fe^{2+}-2G_2(L_{out})+3H]^{5+}$
[i]626.1688	626.1716	$[PD+Zn^{2+}-2G_2(L_{out})+3H]^{5+}$
[h]634.9631	634.9656	$[PD+Ag^{+}-2G_2(Lout)+4H]^{5+}$
[j]645.1818	645.1854	$([PD+Fe^{2+}-G_2(L_{out})+3H]^{5+}$
[i]646.7814	646.7842	$[PD+Zn^{2+}-G_2(L_{out})+3H]^{5+}$
[h]649.4088	649.4127	$[PD-G_1(L_{out})-G_1(K_{out})+4H]^{4+}$
[h]655.5758	655.5809	$[PD+Ag^{+}-G_2(L_{out})+4H]^{5+}$
[i]696.4081	696.4112	$[PD+Zn^{2+}-G_1(K_{out})2G_2(L_{out})+2H]^{4+}$
[i]722.1731	722.1770	$[PD+Zn^{2+}-G_1(K_{out})-G_2(L_{out})+2H]^{4+}$
[h]735.4570, [i]735.4588	735.4642	$[PD-G_1(L_{out})+4H]^{4+}$
[j]745.9398	745.9443	$[PD+Fe^{2+}-G_1(K_{out})+2H]^{4+}$
[h]749.4817	749.4880	$G_0(y)^{+}$
[h]760.4728	760.4801	$[G_{core}(out)-G_2(L_{out})+2H]^{2+}$
[j]777.4586	777.4641	$[PD+Fe^{2+}-G_2(K_{out})-G_2(L_{out})+2H]^{4+}$
[i]789.1632, [j]789.1620	789.1626	$[PD-G_0(K_{out})-G_2(L_{out})+3H]^{3+}$
[h]791.4914, [i]791.4946, [j]791.4927	791.4985	$G_0(L_{out})^{+}$
[h]803.4914, [i]803.4947, [j]803.4930	803.4985	$G_0(K_{out})^{+}$
[h]812.0048, [i]812.0079, [j]812.0057	812.0082	$[G_{core}(out)+2H]^{2+}$
[h]823.5137, [i]823.5175, [j]823.5158	823.5170	$[PD-G_0(K_{out})+3H]^{3+}$

[i]844.1550	844.1596	$[PD+Zn^{2+}-G_0(K_{out})+H]^{3+}$
[h]855.3770	855.3858	$[Ag^{+}+G_0(y)]^{+}$
[h]864.9525	864.9607	$[G_{core}(out)+Ag^{+}+H]^{2+}$
[i]941.9234, [j]941.9198	941.9287	$[PD-G_1(L_{out})-G_2(K_{out})+3H]^{3+}$
[i]962.5613	962.5669	$[PD+Zn^{2+}-G_1(L_{out})-G_2(K_{out})+H]^{3+}$
[i]976.2771, [j]976.2743	976.2832	$[PD-G_1(K_{out})+3H]^{3+}$
[j]994.2496	994.2562	$[PD+Fe^{2+}-G_1(K_{out})+H]^{3+}$
[i]996.9149	996.9213	$[PD+Zn^{2+}-G_1(K_{out})+H]^{3+}$
[i]1062.6676	1062.6755	$[PD-G_0(K_{out})-G_1(K_{out})+2H]^{2+}$
[i]1068.6675	1068.6755	$[PD-G_0(K_{out})-G_1(L_{out})+2H]^{2+}$
